# Anatomical Left Superior Vena Cava Correction: An Option for Left Ventricular Recruitment?

**DOI:** 10.1055/a-2531-3126

**Published:** 2025-03-28

**Authors:** Frederike Bieling, Robert A. Cesnjevar, Michela Cuomo, Annika Weigelt, Sven Dittrich, Ariawan Purbojo

**Affiliations:** 1 Department of Pediatric Surgery, Friedrich-Alexander-University Erlangen/Nürnberg, University Hospital Erlangen, Erlangen, Germany; 2 Department of Pediatric Cardiac Surgery, University Children's Hospital Zurich—Eleonorenstiftung, Zurich, Switzerland; 3 Department of Pediatric Cardiac Surgery, Friedrich-Alexander-University Erlangen/Nürnberg, University Hospital Erlangen, Erlangen, Germany; 4 Department of Pediatric Cardiology, Friedrich-Alexander-University Erlangen/Nürnberg, University Hospital Erlangen, Erlangen, Germany

**Keywords:** aortic valve and root, cardiac anatomy/pathological anatomy, congenital heart disease, echocardiography (all modalities, applications), anatomy, aorta/aortic

## Abstract

**Background:**

Left superior vena cava (LSVC)-related obstruction of mitral inflow is a rare condition in patients with complex cardiac anomalies like hypoplastic left heart complex. We report on the impact of establishing LSVC to right superior vena cava (RSVC) continuity on the growth of borderline hypoplastic left ventricular structures as an addendum to our previously published work.

**Methods:**

Twenty-two patients underwent LSVC to RSVC anastomosis, whereas six had LSVC ligation ( *n* = 4) or clip closure ( *n* = 2), all alongside congenital heart defect correction. The indication was LSVC-related obstruction of left ventricular inflow due to a dilated coronary sinus. Clinical data were systematically reviewed, with regular follow-up. Left ventricular end diastolic diameters (LVEDD), aortic valve diameters, and left ventricular outflow tract (LVOT) diameters were recorded using echocardiography.

**Results:**

Follow-up showed 90% survival at 3.3 ± 0.4 years. Mean LVEDD Z-scores improved from −2.19 ± 0.35 to −1.24 ± 0.26 after repair ( *p* < 0.01) and to −1.33 ± 0.56 at 6-month follow-up. In patients without mitral repair, LVEDD Z-scores improved from −2.11 ± 0.62 preoperatively to −1.85 ± 0.88 postoperatively ( *p* < 0.05). LVOT Z-scores increased from −2.49 ± 0.48 to −0.87 ± 0.75 ( *p* < 0.05) and aortic valve Z-scores improved from −1.08 ± 0.57 to 0.5 ± 0.39 over 24 months ( *p* < 0.05).

**Conclusion:**

Anatomical LSVC correction may improve left ventricular filling and growth of the left ventricle, aortic valve, and LVOT in patients with borderline left ventricles and could be considered without as a potential recruitment strategy.

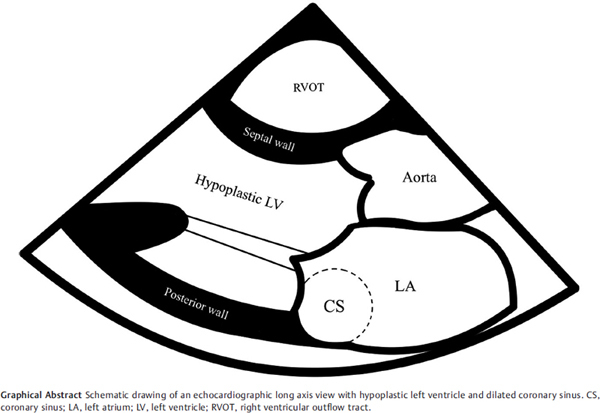

## Introduction

The persistent left superior vena cava (LSVC) is an embryological venous remnant of the left anterior cardinal vein with a prevalence of 0.3% in the healthy population and from 1.3% up to 11% in patients with cardiac anomalies. 1 2 3 4 Well known difficulties related to this anatomical feature are achieving adequate venous drainage during cardiopulmonary bypass, challenges regarding pacemaker lead implantation and central venous catheter placement. 5 The occurrence of an LSVC is thought to be rather hemodynamically irrelevant. Some authors report that an LSVC may be of pathophysiological importance due to its contribution to left ventricular (LV) inflow obstructions in patients with hypoplastic left heart complex (HLHC) and similar constellations. 4 6 7 It is well known that blood flow-directed remodeling is necessary for cardiac morphogenesis and changes in flow dynamics can influence the development of cardiac structures. 8 9 It is conceivable that an inflow obstruction caused by the LSVC can impede LV development and growth. Typically, the left jugular, subclavian, and hemiazygos vein drain into the LSVC before the left atrium is entered close to the left atrial appendage. The LSVC runs transversely in the left atrioventricular groove on the posterior side of the heart then transitions into the coronary sinus and proceeds through the left atrium toward the right atrium. Especially in case of dilatation the coronary sinus protrudes into the left atrium in front of the posterior mitral valve annulus before it drains into the coronary sinus (see central image). 4 Because of multiple empirical reports and its anatomical proximity to the mitral annulus, it must be assumed that especially large-sized LSVCs have a negative impact on mitral inflow and growth. And it is only then that surgical repair has to be evaluated. There have been several surgical attempts to diminish the supravalvular stenosing effect due to the drainage from the LSVC protruding coronary sinus for example, by Cochrane, Kreutzer, and Vargas et al. 10 11 12 13 14

There are only few reports of true anatomical repairs by surgically creating an innominate vein with a direct end-to-side-anastomosis of the mobilized LSVC to the right superior vena cava (RSVC). 15 16 Our group has established this surgical approach for LSVC related obstructions since 2015. 17 The first attempt was successful in a patient with a large LSVC, mitral hypoplasia, smallish left ventricle (LV), a midmuscular ventricular septal defect (VSD), hypoplastic aortic annulus, aortic arch obstruction, and coarctation. The arch obstruction was repaired together with pulmonary artery banding and concomitant LSVC repair. 17 The hypothesis that LSVCs might have a more relevant impact in patients with hypoplastic or borderline-sized LV structures (HLHC or borderline LV) was thereby promoted. 17 We were able to confirm the positive effect of anatomical LSVC repair on substantial mitral valve growth for patients with hypoplastic mitral valves and patients with HLHC in a rather large patient series and published our work in 2021. 17 The positive impact of mitral obstruction relief on mitral growth due to this surgical approach convinced us to pursue further investigations on a possible effect of our anatomical LSVC repair regarding LV, aortic valve (AoV), and left ventricular outflow tract (LVOT) growth retro- and prospective in the meanwhile growing patient group. This study should be seen as an addendum to our previously published work.

## Patients and Methods

### Clinical Setup

The study was approved by our local ethics committee (Ethikkommission der Friedrich-Alexander Universität Erlangen-Nürnberg) and the patients' and guardians' consent was obtained (161_20 Bc). All patients underwent surgical correction of a left superior vena cava in conjunction with repair or palliation of congenital anomalies in our center between April 2015 and July 2020. An interprofessional team consisting of congenital cardiac surgeons and pediatric cardiologists reviewed the clinical data and all acquired diagnostic images systematically for collaborative decision-making. The members indicated the surgery and took care of the patients throughout surgery and postoperatively. Outpatient cardiologists from our institutional network (Netzwerk AHF Nordbayern) provided follow-up care for all patients after discharge and transferred them back if special imaging, such as computed tomography, magnetic resonance imaging, or a diagnostic cardiac catheter became necessary. We used the patients' surgical and cardiological hospital reports for retrospective data collection. Echocardiographic images were reviewed and analyzed by two experienced congenital cardiac surgeons using electronic imaging software with measurement tools (syngoDynamics-workplace, Siemens-Healthcare, Version July 2018). Standardized planes were used for echocardiographic measurements. All parameters were recorded after birth (t0), preoperatively (t1), intraoperative (t2), at time of discharge (t3), 6 months (t4), 12 months (t5), and 24 months (t6) after correction.

### Surgical Procedure

**Fig. 1:**
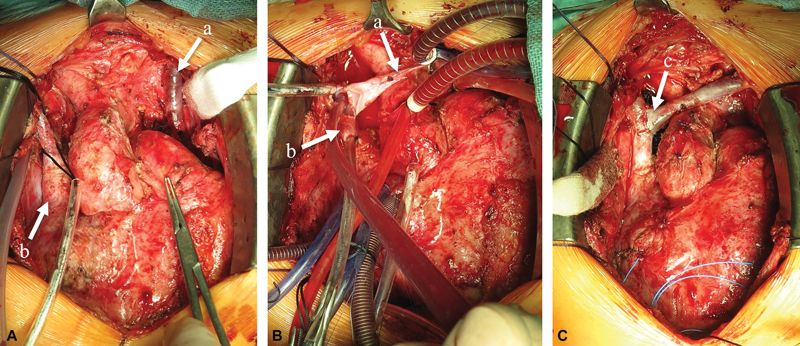
Surgical technique of anatomical LSVC repair. **(A)** mobilized LSVC **(a)** and RSVC **(b)** without bridging vein. **(B)** detached, cannulated and partially anastomosed LSVC **(a)** ; cut open, cannulated and partially anastomosed RSVC **(b)** . **(C)** final result of the LSVC to RSVC end-to-side anastomosis **(c)** . LSVC, left superior vena cava; RSVC, right superior vena cava.

Twenty-two patients underwent anatomical correction by direct end-to-side anastomosis of the LSVC to the right SVC without the use of foreign material, six patients with a small innominate vein underwent anatomical correction by LSVC ligation ( *n* = 4) or clip closure ( *n* = 2) given a sufficiently large LSVC to RSVC connecting vein (vena anonyma) existed. The decision to consider the innominate vein as “sufficiently large” was predominantly based on intraoperative findings by the operating surgeon, complemented by preoperative echocardiographic and cardiac catheterization imaging. Indication for surgery was LSVC related obstruction of LV inflow due to a dilated coronary sinus in conjunction with congenital heart disease, depicted as shown in the patient's characteristics ( Table 1 ). This study focuses exclusively on LSVC with drainage to the coronary sinus, as LSVC draining to the left atrium or other rare variants represent distinct entities. In the absence of a bridging (innominate) vein the LSVC was completely mobilized from the left subclavian vein down to the left atrial appendage by clipping or ligating the vena hemiazygos and all small side branches. After standard heparinization and arterial cannulation, the left and the right vena cava superior as well as the inferior vena cava were cannulated before detaching the LSVC from its atrial connection. During cardioplegic arrest the mitral valve (23) or atrioventricular valve (5) was just inspected and sized ( *n* = 28), repaired ( *n* = 12), or, if necessary, replaced ( *n* = 4) and the subvalvar apparatus was inspected. The due to the drainage of the LSVC enlarged coronary sinus was unroofed and the LSVC was resected at the cardiac level. The proximal stump was closed with a 6 × 0 polypropylene suture (Prolene, Ethicon). At the end of the procedure the LSVC was end-to-side anastomosed to the RSVC with a 7 × 0 running polypropylene suture (Prolene, Ethicon) ( Fig. 1 ). This last part was performed on bypass with the heart beating during rewarming of the patient. To avoid contraction of the anastomosis in the form of a purse–string effect the suture was interrupted on two opposite ends. Near-infrared spectroscopy S-monitoring, central venous pressure measurements, and echocardiography were performed to check anastomotic function. When stenosis was suspected or postoperative assessments before next surgical steps needed to be done the patients underwent postoperative angiocardiography ( *n* = 16). No anticoagulation was administered postoperatively due to the LSVC correction. Patients who underwent additional procedures that required anticoagulation were anticoagulated according to standard guidelines.

**Table 1:** Patient diagnosis

Patient	Sex	Age (mo)	Diagnosis	Primary repair	Outcome	Status	Patency	Follow-up (y)	RACHS	Aristotle score
1	F	4	Unbalanced AVSD	Yes	Biventricular repair	Alive, stent in RSVC	Yes	2.82	3	9
2	M	5	Unbalanced AVSD	Yes	Biventricular repair	Alive	Yes	4.48	3	9
3	M	7	AVSD and coarctation, smallish LV	Yes	Biventricular repair	Alive	Yes	3.93	3	9
4	M	28	Unbalanced AVSD	Yes	Biventricular repair	Alive	Yes	2.11	6	15
5	F	29	AVSD, hypoplastic aortic valve, PAPVD, smallish LV, left isomerie	Yes	Biventricular repair, LV reconstruction	Alive	Yes	2.42	3	8
6	M	39	AVSD, common atrium, congenital complete heart block	No	Biventricular repair	Alive	Yes	1.22	3	9
7	M	57	AVSD, smallish LV	No	Biventricular repair, MV replacement	Alive	Yes	8.09	3	8
8	M	0,06	HLHC, hypoplastic aortic valve, hypoplastic aorta, coarctation, mitral hypoplasia	No	Biventricular repair	Died at the age of 9 d due to low cardiac output	Yes	4.96	6	15
9	M	0.1	HLHC, M. Shone, hypoplastic mitral valve, hypoplastic aortic arch and coarctation	No	Biventricular repair	Alive	Yes	1.43	6	15
10.	F	0.2	HLHC, hypoplastic aortic arch, coarctation, mitral stenosis and VSD	No	Biventricular repair	Alive	Yes	3.14	6	15
11.	M	0.5	HLHC, hypoplastic aortic arch, hypoplastic aorta, coarctation, mitral stenosis	Yes	Failed biventricular repair converted to reverse shunt (Pott shunt) and bilateral PA-banding	Died before reconversion	Yes	0.82	6	15
12.	F	1	HLHC, mitral stenosis, supravalvular membrane	Yes	Biventricular repair	Alive	Yes	0.08	3	8
13.	F	2	HLHC, multiple VSDs, Cor triatriatum, mitral hypoplasia, smallish aorta	Yes	Biventricular repair	Alive	Yes	2.52	3	8
14.	F	4	HLHC, hypoplastic aortic arch, coarctation, hypoplastic aortic valve, mitral hypoplasia	No	Biventricular repair	Alive	Yes	1.82	6	15
15.	M	7	HLHC, M. Shone, hypoplastic aortic arch, coarctation, hypoplastic mitral valve, supramitral membrane	No	Biventricular repair	Alive	Yes	1.46	6	15
16.	M	11	HLHC, M. Shone, hypoplastic mitral valve, LVOTO, hypoplastic aortic arch, coarctation, arteria lusoria	Yes	Biventricular repair	Alive	Yes	1.43	6	15
17.	M	39	HLHC, aortic atresia, hypoplastic aortic arch, and VSD	Yes	Biventricular repair (Mustard–Rastelli)	Alive	Yes	6.24	4	11
18.	M	45	HLHC, Shone-complex, s.p. repair of aortic arch hypoplasia and coarctation, mitral stenosis	Yes	Biventricular repair MV replacement	Alive	Yes	8.95	4	11
19.	M	0,5	DORV, pulmonary atresia	Yes	Biventricular repair	Alive	Yes	4.48	3	7,5
20.	F	6	DORV-Fallot-type, smallish LV	Yes	Biventricular repair	Alive	Yes	3.33	2	8
21.	M	13	Heterotaxia, TAPVD, DORV, PA	No	Biventricular repair (Mustard–Rastelli)	Died 1.5 years repair	Yes	1.70	4	11
22.	F	14	DORV, hypoplastic mitral valve and hypoplastic LV, supramitral membrane	No	Still awaiting biventricular repair after LSVC rerouting	Alive	Yes	1.88	3	8
23.	M	26	DORV, hypoplastic mitral valve, PS	No	Biventricular repair	Alive	Yes	2.22	3	10,3
24.	F	3	TAC type 1, mitral hypoplasia, supravalvar membrane	No	Biventricular repair	Alive	Yes	8.95	4	11
25.	F	4	Cor triatriatum, smallish LV	No	Biventricular repair	Alive	Yes	7.77	3	8
25.	F	6	TGA,VSD, PS, smallish LV	Yes	Biventricular repair	Alive	Yes	2.69	4	11
27.	M	11	VSD, mitral stenosis, supravalvular membrane	Yes	Biventricular repair	Alive	Yes	4.81	3	8
28.	M	11	VSD, mitral stenosis, Down's syndrome	Yes	Biventricular repair	Alive	Yes	0.99	3	8

Abbreviations: AVSD, atrioventricular septum defect; DORV, double-outlet right ventricle; F, female; HLHC, hypoplastic left heart complex; LV, left ventricle; LVOTO, left ventricular outflow tract obstruction; LSVC, left superior vena cava; M, male; MV, mitral valve; PA, pulmonary artery; PAPVD, partial anomalous pulmonary venous drainage; PS, pulmonary stenosis; RACHS, risk adjustment for congenital heart surgery; TAC, truncus arteriosus communis; TAPVD, total anomalous pulmonary venous drainage; TGA, transposition of the great arteries; VSD, ventricular septal defect

Notes: Biventricular repair was either VSD repair and mitral reconstruction (RACHS 3, Aristotle score 8), AVSD repair (RACHS 3, Aristotle score 9), biventricular repair of HLHC (RACHS 6, Aristotle score 15), Ross–Konno Operation (RACHS 4, Aristotle score 11), or repair of complex transposition (RACHS 4, Aristotle score 11).

Additional procedures were mitral ( *n* = 7) or atrioventricular ( *n* = 5) valve surgery; right ventricle to pulmonary artery conduit ( *n* = 4); first-stage palliation ( *n* = 2), biventricular repair of HLHC ( *n* = 7), mitral valve replacement ( *n* = 4), and tetralogy of Fallot repair ( *n* = 1).

### Echocardiography

**Fig. 2:**
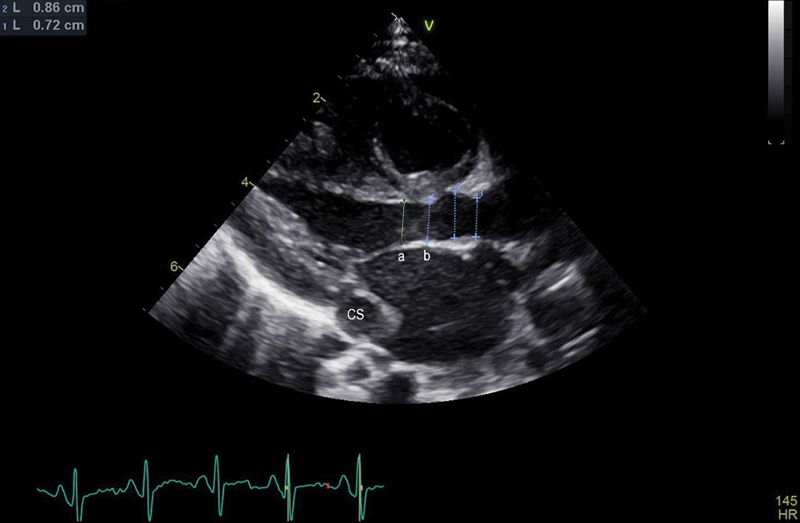
Long-axis view before anatomical left superior vena cava repair. ( **A** ) LVOT diameter, ( **B** ) AoV diameter. AoV, aortic valve; CS, coronary sinus; LVOT, left ventricular outflow tract.

Left ventricular end diastolic diameters (LVEDD), AoV diameters, and LVOT diameters were measured pre-, intra- and postoperatively by 2D-echocardiography (GE Vivid E95 and Vivid 7, GE Healthcare; Philips Affinity 50G, Royal Philips Healthcare Company) in two standard planes (long-axis and four-chamber view) with the left-sided AoV open in diastole ( Fig. 2 ). Intraoperative measurements were made by transesophageal echocardiography. Changes were expressed by either comparing the development to the individual norm values or by using Z-scores to express LV and LVOT hypoplasia, growth, and development. 18 19 20 Each patient served as his own control.

### Follow-up

Follow-up after surgery was 2.3 ± 0.45 years. Most of the patients underwent staged repair. Previously palliated patients underwent definitive repair and therefore had a complete diagnostic workup (including, cardiac catheterization). All patients were included in a regular institutional follow-up.

### Statistical Analysis

Statistical software (EXCEL, Microsoft, and SPSS for Windows, Microsoft Corporation, Redmond,

Washington State, United States) was used for analysis. Values are expressed with standard deviation of the mean. Student's *t* -test or the nonparametric U-test were used according to the nature of the tested variables. Statistical significance was assumed at a probability value of less than 0.05 ( *p* < 0.05).

## Results

Twenty-eight patients (median age: 0.46 years; range: 2 days to 4.75 years) were included in the study.

Patients' characteristics and diagnosis are presented in Table 1 .

In total three patients died postoperatively. There was no early mortality after LSVC to RSVC end-to-side-anastomosis and one early death after LSVC clip closure. This patient with HLHC died at the age of 9 days, 7 days after surgery due to multiorgan failure caused by low cardiac output due to the massively stenosed aortic arch.

Two patients died late during follow-up. Both were included in our study until the 6-month follow-up. One of them with HLHC died at the age of 10 months, after failed primary biventricular repair. Following initial aortic arch repair and after LSVC to RSVC end-to-side-anastomosis, a rescue procedure had to be performed due to postoperative low cardiac output with multiorgan failure. Nine months after hospital discharge the meanwhile renal insufficient and ventilated patient arrived in our hospital due to septic shock, which ultimately caused his death.

The second one with heterotaxia and pulmonary atresia died in an external pediatric hospital 9 months after successful biventricular repair due to a fulminant septic event, possibly related to pulmonary valve endocarditis of his right ventricle to pulmonary artery conduit. Despite the unusual rapid deterioration of the patient, no autopsy was performed by request of the parents.

Follow-up was complete with 90% survival after 2.3 ± 0.45 years. In this time period, 57% of the patients ( *n* = 16) underwent cardiac angiography for different indications. In three patients, angiography was indicated due to issues concerning the LSVC. One of them with unbalanced atrioventricular septal defect (AVSD) who underwent biventricular repair had a significantly stenosed RSVC below the LSVC–RSVC anastomosis at the level of cannulation and was therefore balloon-dilated and later stented. Reintervention had to be done twice and the RSVC still remained patent 4 years after surgery. Another patient had a stenosed RSVC and LSVC, that needed to be dilated but stayed patent since then. Neither of the patients needed long-term systemic anticoagulation treatment. The third patient developed a marginal thrombus formation on the RSVC directly proximal to the anastomosed LSVC, which dissolved spontaneously during follow-up. In all other angiographies the LSVCs presented patent. No additional surgery was needed due to LSVC-related complications.

Two patients underwent mitral valve replacement in conjunction with LSVC-correction. One patient with HLHC, Shone complex, and previous palliative repair of aortic arch hypoplasia and coarctation underwent mitral and AoV replacement ( Table 1 ). Another patient with AVSD and smallish LV underwent mitral valve replacement due to unrepairable mitral valve regurgitation after initial AVSD repair.

**Fig. 3:**
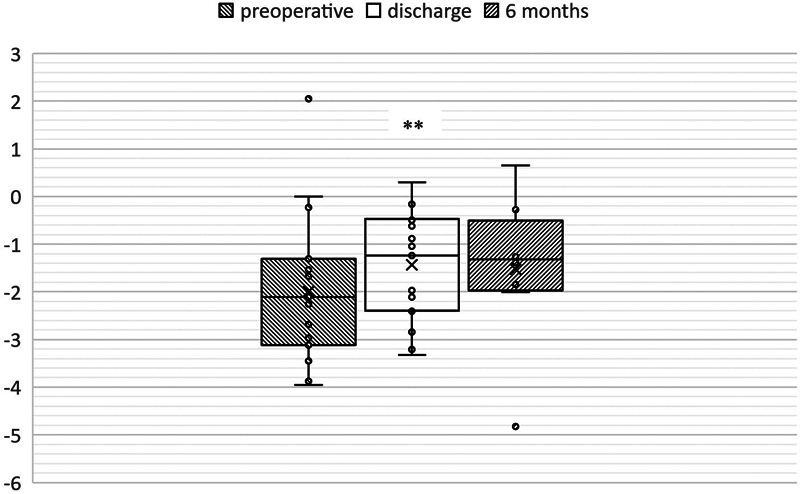
Changes of LVEDD Z-scores. Analysis of LVEDD Z-scores preoperatively (t1), at time of discharge (t3) and 6 months after the operation (t5). Between t1 and t3 LVEDD Z-scores changed significantly (** *p* < 0.01). LVEDD, left ventricular end diastolic diameter.

**Fig. 4:**
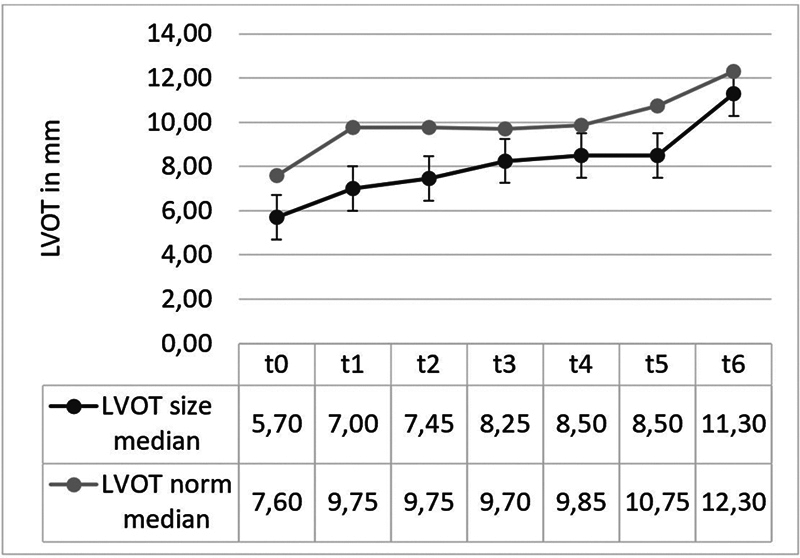
Median development of the left ventricular outflow tract (LVOT) diameter. Comparison of the median LVOT diameter to the median norm value for the LVOT diameter of this patient group in absolute numbers. An approximation of the median LVOT diameter to the median norm LVOT diameter is visible. t0: after birth, 21 patients; t1: preoperative, 20 patients; t2: intraoperative, 19 patients; t3: at time of discharge, 19 patients; t4: 6 months, 6 patients; t5: 12 months after correction, 5 patients; t6: 24 months after correction, 8 patients.

**Fig. 5:**
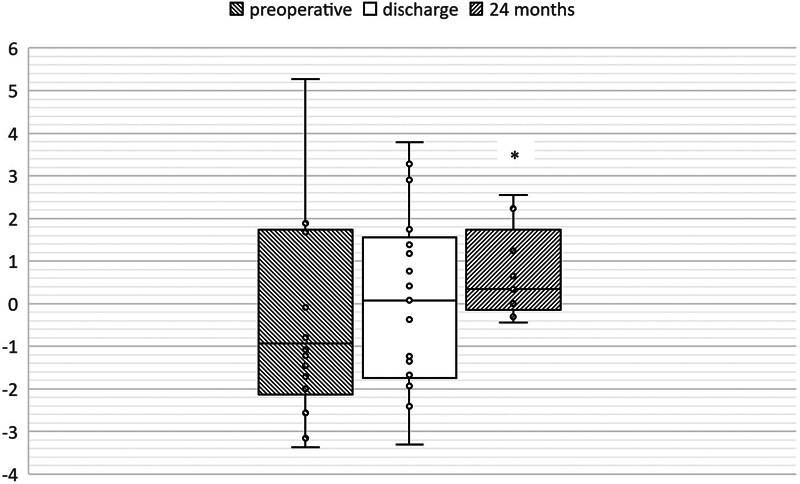
Changes of AoV diameter Z-scores. Analysis of aortic valve diameter Z-scores preoperatively (t1), at time of discharge (t2) and 24 months after the operation (t6). Between t1 and t6 AoV diameter Z-scores changed significantly (* *p* < 0.05). AoV, aortic valve.

Mean LVEDD Z-scores were −2.19 ± 0.35 (range: −3.96 to 2.05) and increased to −1.24 ± 0.26 (range: −3.33 to 0.3) immediately after repair ( *p* < 0.01). In further follow-up investigations Z-scores remained unchanged around −1.33 ± 0.56 (range: −4.83 to 0.65) after 6 months and −0.92 ± 0.39 (range: −3.37 to 0.43) after 24 months ( Fig. 3 ). LVOT Z-scores increased significantly from −2.49 ± 0.48 to −0.87 ± 0.75 in the 24-month follow-up ( *p* < 0.05) ( Fig. 4 ). AoV diameter Z-scores changed from −1.08 ± 0.57 to 0.5 ± 0.39 between preoperative measurements and 24-month follow-up ( *p* < 0.05) ( Fig. 5 ). The subgroup of patients with a small bridging vein who received LSVC ligation ( *n* = 4) or clip closure ( *n* = 2) developed similarly to the group who received anatomical correction.

To exclude potential influencing factors of concomitant mitral surgery, a subgroup of 12 patients without mitral surgery was analyzed further. Results of LVEDD Z-scores, LVOT Z-scores and AoV diameter Z-scores are summarized in Table 2 . LVEDD Z-scores changed significantly between preoperative (t1: −2.11 ± 0.62; range: −3.78 to −0.23) and postoperative measurements (t3: −1.85 ± 0.88, range: −4.83 to 0.65, *p* < 0.05).

**Table 2:** Z-scores of patients without concomitant mitral valve repair ( *n* = 12) in comparison between preoperative examination and time of discharge after surgical repair

	t1 (time of preoperative setting)	t3 (time of discharge)	*p* -Value
LVOT (median [range])	−2.46 (−5.36 to −1.39)	−0.40 (−4.28 to 2.89)	
AoV (median [range])	−1.22 (−2.56 to 5.27)	0.80 (−3.30 to 3.79)	
LVEDD (median [range])	−2.27 (−3.78 to −0.23)	−1.86 (−3.21 to −0.16)	<0.05

Abbreviations: AoV, aortic valve; LVEDD, left ventricular end diastolic diameter; LVOT, left ventricular outflow tract.

## Discussion

Even though LSVC is a rare finding in the healthy population and generally considered an anatomical feature with no pathophysiological relevance, there are significant associations between its occurrence and other malformations. Buirski et al state that the “persistent left SVC is especially frequent in association with common AVSDs, double-outlet right ventricle and situs abnormalities” and has found an LSVC incidence of 6.9% in children with HLHC. 2

It is well known that deviating cardiac flow patterns are suspected to have an impact on cardiac development and growth and can contribute to the genesis of HLHC. 8 It has been shown echocardiographically that LV hypoplasia evolves in severity with gestation. 21 22 Regarding these findings Hickey et al conclude that normal cardiac morphogenesis necessitates not only intrinsic patterning but also blood flow-directed remodeling. Blood flow remodeling refers to the secondary development and differentiation of structures as a direct consequence of shear stress and flow dynamics within them. 8 A dilated coronary sinus caused by an LSVC can lead to massive mitral inflow obstruction and therefore strongly modified shear stress and flow dynamics. 14 23 Experimental obliteration of blood flow in the LV of 5-day-old chicken embryos results in varying degrees of LV hypoplasia, which strongly supports the hypothesis that inflow obstruction contributes to a by the authors so called “flow-volume hypoplasia.” 9 Besides inflow obstructions an atrial septal defect (ASD), AVSD, and especially an unbalanced AVSD can lead to abnormal flow patterns. Emani et al have shown that ASD restriction in children with borderline LVs is predictive of increases in LV filling and growth. 24 Our study cannot make any statement concerning the embryological development of HLHC. Nevertheless, this pathophysiological background and the recruitment strategies that have already proved helpful brought us to the idea that normalizing mitral and caval blood flow by an anatomical correction of the LSVC in conjunction with other LV recruitment strategies could promote LV filling and growth. 25

The results of the postnatal anatomical LSVC correction in our cohort presented as following.

Total LVEDD Z-scores increased significantly between preoperative measurements and time of discharge ( *p* < 0.01). After exclusion of patients with concomitant mitral repair, changes of LVEDD Z-scores remained significant( *p* < 0.05). Since this enlargement of LVEDD Z-scores occurred in such a brief period of time, it can be assumed that it is primarily caused by dilatation due to the enhanced mitral inflow rather than growth. The Z-scores remained the same or increased insignificantly in the follow-ups ( Fig. 5 ). Z-scores of the LVOT and AoV diameter demonstrated enlargement in subsequent follow-up assessments, failing to reach statistical significance in the comparison of pre- and postoperative measurements ( Figs. 4 , 5 ). This correlation suggests that the sustained increase in LV inflow and filling might promote growth in LVOT and AoV, potentially indicating that the persistent enlargement of LVEDD could be attributed to growth rather than dilatation.

In comparison to other LSVC correction procedures like intra-atrial size reduction connection to the RSVC via Gore-Tex® graft or end-to-end-anastomosis with the right atrial appendage, our proposed anatomical correction seems to provide the most normal blood flow conditions. 10 12 14 Additional benefits of the anatomical repair are the growth potential, which is not given in the graft approach, and the possibility of performing the procedure during rewarming and therefore shortening time of ischemia, which is not possible in intra-atrial size reductions.

The presented study faces limitations within its design, which might restrict the value of our results. Primarily, indication for LSVC repair remains to be conjunct to other concomitant cardiac malformations. As our cohort consists therefore of children with a rather wide range of cardiac malformations, it would be interesting to evaluate what impact certain associated defects have on the postoperative development of LV structures. ASDs, AVSDs, and especially unbalanced AVSDs in conjunction with an LSVC could be of particular interest as a dilated coronary could aggravate the left-to-right shunt in this constellation by diverting the blood flow through the septal defect. In this setting, a greater dimension of LV growth is plausible as ASD correction itself correlates with increased LV dimensions in patients with borderline LVs. Moreover, appropriate mitral valve repair has been shown to promote the growth of LV structures in patients with borderline LVs. 26 27 We tried to overcome this influence by analyzing the subgroup of patients without having received mitral valve repair ( *n* = 7), mitral valve replacement ( *n* = 4), or atrioventricular surgery ( *n* = 5) alongside LSVC correction. We were able to still confirm statistical significance but based on the analysis of this small subgroup within a small population, these results have to be interpreted with caution. The definitive attribution of the observed growth in LV structures to these additional procedures remains therefore uncertain and one might postulate that influence of different surgical repairs might be the cause for improvement of Z-scores. However, based on a previous study at our center which found a positive effect of LSVC correction on mitral valve growth and the fact that the subgroup of patients without concomitant mitral surgery demonstrated significant changes in LVEDD Z-scores as well, we posit that the impact of mitral valve repair combined with LSVC correction could be synergistic. 17

Secondarily, similar effects might be discussed based on the small population with a wide age range.

It is still to evaluate if age at time of the correction has an impact on the growth potential of LV structures and beyond that, if there may be some hyperplastic potential left in younger children.

To gain knowledge in this regard, larger patient cohorts should be evaluated and a multicentre trial would be the next step to compare different approaches to large LSVCs in patients with borderline LVs. It would therefore be important to establish criteria for deciding whether or not to perform anatomical correction of LSVC. Echocardiographic measurement of the diameter of the coronary sinus or assessment of the LSVC diameter by echocardiography or angiography would be conceivable. Should the LSVC correction as a recruitment strategy in biventricular settings prove successful in further studies in the future, anatomical LSVC repair might also be considered as a staged recruitment strategy in children with initial single ventricle palliation like other inflow and outflow tract relieving procedures, which have already been evaluated.

## Conclusion

We report our results observing the development of LV structures after LSVC to RSVC end to side anastomosis, LSVC ligation or clip closure in conjunction with correction or palliation of other cardiac anomalies in children with borderline LVs, as an addendum to our previously published work. Within a heterogeneous population, including concomitant mitral valve repair, we observed an increase in LV filling and growth, as well as significant growth of the AoV and LVOT and were able to confirm improved LVEDD Z-scores in the subgroup of patients without mitral surgery. No relevant LSVC-related complications were recorded.

In summary, these results might give a hint that an anatomical LSVC correction could be considered a recruitment strategy in borderline LVs with biventricular approach on a case-by-case basis, especially if a mitral inflow obstruction due to the LSVC is observed and should be validated in larger population studies with homogeneous subgroups.
